# Supplementary material for: The Soybean bZIP Transcription Factor Gene GmbZIP2 Confers Drought and Salt Resistances in Transgenic Plants
Source: Int J Mol Sci. 2020 Jan 20;21(2):670. doi: 10.3390/ijms21020670 (PMC7013997; doi:10.3390/ijms21020670)
Supplement: Supplementary file 1 [file ijms-21-00670-s001.zip › ijms-677901 - supplementary.pdf]

**Supplemental Figure S1.** Phylogenetic tree analysis of 15 soybean bZIPs.

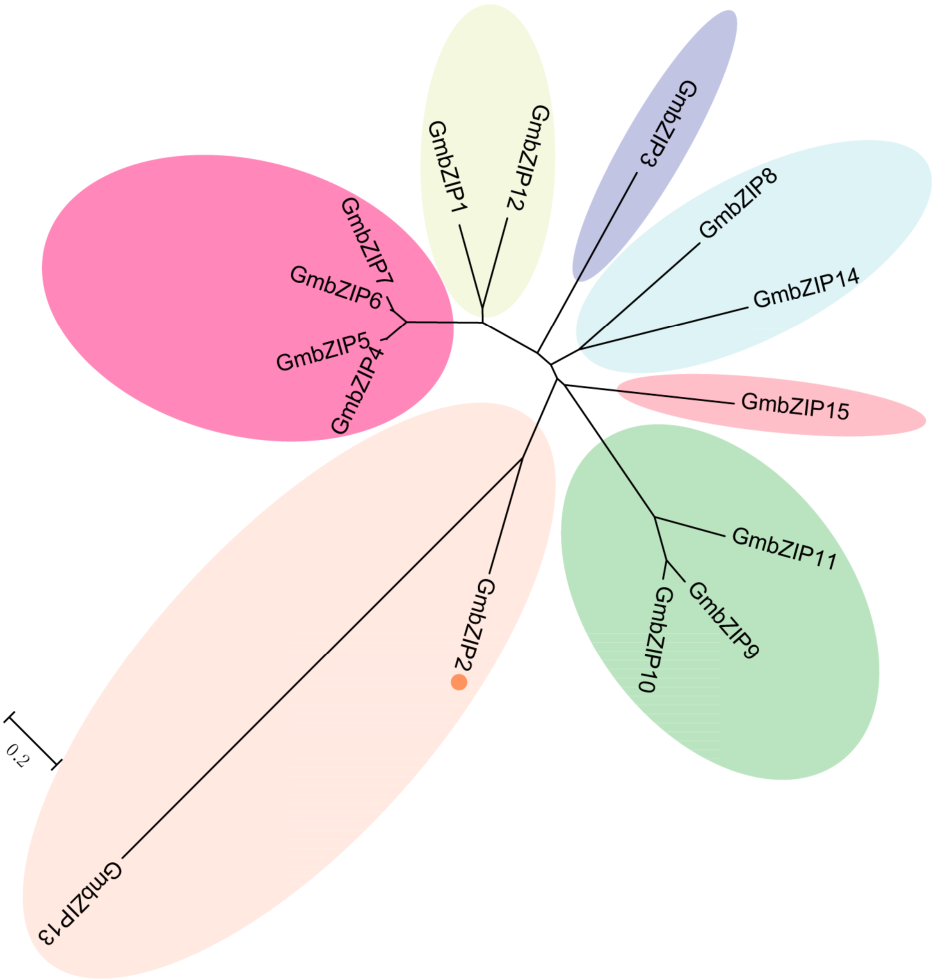

**Supplemental Figure S2.** Multiple Sequence Alignment of 15 soybean bZIPs.

Leucine zipper region

```
GmbZIP1    LKRQRRKQSNRESARRSRLRKQAECDELAQRAEALKEENAT.LRSEVSQIRSEYEQLRSENAALKERLGDIPGVATPG.KEDLRSG
GmbZIP2    ERKLRRMQSNRESARRSRYRKKQHIENLTSQLNRLRIQNRL.LKNQLASTMHQNLLLSLHNDHLKSESVALMATLSDL.CGILGTM
GmbZIP3    ERKRKRMESNRESARRSRMKKQKQLEDLTDEVSRLEGENAR.LAPSIKVKEEAYVEMEAANDILRAQTMELADRLRFL.NSIIEIA
GmbZIP4    LKRERRKQSNRESARRSRLRKQAETEELARKVESLNAENAT.LKSEINRLTESSEKMRVENATLRGKLKNAQLGQTQ..EITLKII
GmbZIP5    LKRERRKQSNRESARRSRLRKQAETEELARKVESLNAENAT.LKSEINRLTESSEKMRVENATLRGKLKNAQLRQTQ..EITLNII
GmbZIP6    LKRERRKQSNRESARRSRLRKQAETEELARKVEMLTAENVS.LKSEITRLTEGSEQMRMENSALREKLINTQLGPRE..EITLSSI
GmbZIP7    LKRERRKQSNRESARRSRLRKQAETEELARKVDMLTAENVS.LKSEIIQLTEGSEQMRMENSALREKLRNTQLGQRE..EIILSSI
GmbZIP8    ERRLRRKIKNRESAARSRARKQAYHNELVSKVSRLEEENVK.LK.KEKLCCYVHKAIFVYMSLLKITFNNKEVQRLKI.VAD....
GmbZIP9    PKRAKRMLANRQSAARSKERKIRYTSELEKKVQTLQTEATN.LSAQLTMLQRDTTDLTAQNKELKLRLQAFEQEAQL..REDLNEA
GmbZIP10   PKRAKRILANRQSAARSKERKIRYTSELERKVQTLQTEATN.LSAQLTMLQRDTTDLTTENKELKLRLEALEQEAQL..REDLNEA
GmbZIP11   PKRAKRILANRQSAARSKERKMRYISELEHKVQTLQTEATT.LSAQLTLLQRDSAGLTNQNSELKFRLQSMEQQAKL..RDALNEA
GmbZIP12   LKKQKRKQSNRESARRSRLRKQAECEELQKRVESLGSENQT.LREELQRVSEECKKLTSENDSIKEELERLCGPEAV..ANLE...
GmbZIP13   LSGMWKTPAERCFIWLGGFRSSELLKIVRNQLEPLTEQQLMGIYNLQQSSQQAEDALSQGMEALQQSLSETLSSSSLGPSGSENVA
GmbZIP14   LQKQRRMIKNRESAARSRERKQAYTVELESLVTHLEEEN.......AILLKQEADRKRQRFNQLMECLIPVEEKRKP..KPMLRRV
GmbZIP15   VKRVRRMLSNRESARRSRRRKQAHLTDLETQVSQLRGENST.LLKRLTDVSQKYSDSAVDNRVLKADVETLRAKVKMA.EETVKRI
Consensus            r                       l
```

**Supplemental Table S1.** RNA-seq data analyses of 15 soybean bZIPs.

| Gene | Gene ID | CK_treat-Expression | GH_treat-Expression | log2FoldChange(GH_treat/CK_treat) | P-value |
|---|---|---|---|---|---|
| GmbZIP1 | Glyma.08G265600.1 | 351 | 763 | 2.21661 | 0.00011 |
| GmbZIP2 | Glyma.06G048500.1 | 2 | 82 | 1.94359 | 0.03354 |
| GmbZIP3 | Glyma.02G012700.1 | 1195 | 10915 | 1.65979 | 5.20E-05 |
| GmbZIP4 | Glyma.03G255000.1 | 0.4 | 714 | 3.016 | 3.48E-25 |
| GmbZIP5 | Glyma.19G252600.1 | 433 | 5979 | 1.84171 | 4.13E-05 |
| GmbZIP6 | Glyma.16G029000.1 | 572 | 5630 | -0.2974 | 0.01545 |
| GmbZIP7 | Glyma.07G060400.1 | 124 | 1339 | 1.56869 | 0.01356 |
| GmbZIP8 | Glyma.13G075000.1 | 52 | 310 | 1.53616 | 4.35E-15 |
| GmbZIP9 | Glyma.16G168400.1 | 105 | 611 | 2.03963 | 1.05E-32 |
| GmbZIP10 | Glyma.02G082800.1 | 301 | 1401 | -5.8278 | 0.02356 |
| GmbZIP11 | Glyma.12G036400.1 | 1854 | 7022 | 1.16739 | 1.53E-17 |
| GmbZIP12 | Glyma.11G065000.1 | 1009 | 3647 | 2.16598 | 0.00957 |
| GmbZIP13 | Glyma.20G113600.1 | 515 | 1567 | 1.13623 | 2.16E-09 |
| GmbZIP14 | Glyma.08G183600.1 | 53 | 140 | -0.1335 | 0.01786 |
| GmbZIP15 | Glyma.10G162100.1 | 620 | 1621 | -0.4348 | 3.86E-25 |

**Supplemental Table S2.** Putative *cis*-acting elements in the 15 soybean bZIP promoters

| Gene | ABRE | DRE | LTR | MYB | MYC | CGTCA-motif | TGACG-motif |
|---|---|---|---|---|---|---|---|
| GmbZIP1 | 4 | 0 | 0 | 10 | 16 | 0 | 0 |
| GmbZIP2 | 10 | 2 | 1 | 5 | 16 | 1 | 3 |
| GmbZIP3 | 1 | 0 | 2 | 33 | 10 | 1 | 5 |
| GmbZIP4 | 13 | 0 | 0 | 14 | 14 | 2 | 3 |
| GmbZIP5 | 2 | 0 | 0 | 13 | 8 | 0 | 2 |
| GmbZIP6 | 4 | 0 | 0 | 12 | 10 | 1 | 2 |
| GmbZIP7 | 15 | 0 | 0 | 19 | 14 | 2 | 3 |
| GmbZIP8 | 0 | 2 | 0 | 9 | 2 | 0 | 0 |
| GmbZIP9 | 2 | 0 | 1 | 20 | 30 | 0 | 0 |
| GmbZIP10 | 4 | 3 | 3 | 29 | 12 | 1 | 2 |
| GmbZIP11 | 3 | 0 | 2 | 27 | 4 | 1 | 1 |
| GmbZIP12 | 6 | 0 | 1 | 24 | 10 | 0 | 2 |
| GmbZIP13 | 0 | 0 | 1 | 15 | 8 | 0 | 0 |
| GmbZIP14 | 3 | 1 | 1 | 14 | 10 | 0 | 0 |
| GmbZIP15 | 0 | 0 | 1 | 13 | 22 | 1 | 1 |

**Supplemental Table S3.** Functions of elements in the 15 soybean bZIPs promoter.

| Elements | Core sequence | Function |
|---|---|---|
| ABRE | ACGTG/CGCACGTGTC/AACCCGG | ABA- and drought-responsive element |
| DRE | RCCGAC | ABA- and drought-responsive element |
| LTR | CCGAAA | Low-temperature responsiveness element |
| MYB | C/TAACNA/G | Drought responsive element |
| MYC | CANNTG | ABA- and drought-responsive element |
| CGTCA-motif | CGTCA | MeJA responsiveness element |
| TGACG-motif | TGACG | MeJA-responsiveness element |

**Supplemental Table S5.** Primers used for gene expression analysis and vector construction

| Primer | F(5’ to 3’) | R(5’ to 3’) |
|---|---|---|
| GmbZIP1-qRT | CCGTACGACCTTCCCATTGT | AAGGCGAATTTGTGTCCCCA |
| *GmbZIP2*-qRT | GAACCTCACGAGCCAACTGA | AGGGCAACGGATTCGGATTT |
| *GmbZIP3*-qRT | GATCGTCGGAGGGAGGAGAT | GCTCACTTCGTCGGTTAGGT |
| *GmbZIP4*-qRT | TCAGGCGAATCAGACCAACC | GTGAGGAGCGTGACCAGAAG |
| *GmbZIP5*-qRT | TTCAGGTATGACCACCACGC | TTTCCGCCTCTCACGTTTCA |
| *GmbZIP6*-qRT | GTTCCAGCGACGGAAGTGAT | AGTCTCTTTGGAAGCCGGAC |
| *GmbZIP7*-qRT | GGTTACAGCGACGGAAGTGA | AGTCTCTTTGGAAGCCGGAC |
| *GmbZIP8*-qRT | ATGCTCCCGATGCGTATGAG | ACGGGAAACCTTGCTAACCA |
| *GmbZIP9*-qRT | CGAGCTTCTCCTCTTCGACC | GAATCCACCGACAGACTCCG |
| *GmbZIP10*-qRT | AAGTTCATCGGAAAGCCGCC | CGAAGAGGAGAAGGTCGTCG |
| *GmbZIP11*-qRT | TCGGCATTTCCGTCAGACAA | AAGCCTCTGCATCGCTTCTT |
| *GmbZIP12*-qRT | ATATGTGGGGAAGCCAGCAC | ACCATTCTGTGTGGTGCTCA |
| *GmbZIP13*-qRT | GTCGAGCAATGTGGCTGTTG | TCTCAACATGCTGGTTCCCC |
| *GmbZIP14*-qRT | AGACATGGCATCGTCCAAGG | GCGTCGTTTTCGGTCAACAA |
| *GmbZIP15*-qRT | CGGGCCTCTCCTTTTGTGTA | CGGCCAGAAATGATCGGAGA |
| *GmbZIP2* | ATCTTTGCATTTGAACCAAGCC | CTCTTCCCCTACCTCGATGC |
| *GmbZIP2*-pCMBIA1302 | GGGACTCTTGACCATGATGTTTTTCCACCAA | TCAGATCTACCCATGGATGTGAAAGCATGGT |
| *Actin* (*U60506*） | ACATTGTTCTTAGTGGTGGCT | CTGTTGGAAGGTGCTGAG |
| *GmDHN15*-qRT | TTTTGTTTTGTTGTATTGTGTAG | GAAAAATCCTCCACCTGACGA |
| *GmWD40*-qRT | TGCCAGTCTCGTTAGGCTTTTC | CTTATTGAGTTGTTGTTTGGCAG |
| *GmMYB48*-qRT | AACAACACTCTTCAGCCAGTTT | GGGCAAAACAAACTTTCCTCAT |
| *GmGST1*-qRT | CACAATGAGCAGCCCATAGCA | CTTCAACATTCTTCTCACGCTCT |
| *GmLEA*-qRT | GGTGGGTGAAACCGCACAAGA | ATGGATGCCGCCACTCCGCCAG |

**Supplemental Table S6.** RNA-seq data analyses of 5 stress response genes.

| Gene | Gene ID | CK_treat-Expression | GH_treat-Expression | log2FoldChange(GH_treat/CK_treat) | Up/Down-Regulation |
|---|---|---|---|---|---|
| GmDHN15 | Glyma.11G149900 | 3.62041432 | 46354.88544 | 13.64427896 | up |
| GmWD40 | Glyma.15G129100 | 1.33916319 | 3267.956744 | 11.25284139 | up |
| GmMYB48 | Glyma.15G041100 | 23.43817295 | 3904.360652 | 7.380082399 | up |
| GmGST1 | Glyma.07G139800 | 39.2812351 | 31508.76997 | 9.647697427 | up |
| GmLEA | Glyma.U018200 | 0.34561815 | 82.41961276 | 7.897664872 | up |

**Supplemental Table S7.** The number of cis-acting elements that could be bound by basic leucine zipper (bZIP) transcription factors in 5 stress response genes promoter.

| Gene | ACGT |
|---|---|
| GmDHN15 | 21 |
| GmWD40 | 12 |
| GmMYB48 | 16 |
| GmGST1 | 8 |
| GmLEA | 10 |
